# Fatal leukodystrophy in Costello syndrome: a case report

**DOI:** 10.1186/s12887-023-04166-z

**Published:** 2023-07-24

**Authors:** Virgilio E. Failoc-Rojas, Piero A. Quiroz Ugaz, Dante A. Loconi León, Sandra Zeña-Ñañez

**Affiliations:** 1 Facultad de Medicina, Universidad Cesar Vallejo, Raúl Mata La Cruz s/n, Piura, 20001 Perú Peru; 2 Universidad Nacional Pedro Ruiz Gallo, 391 Juan XXIII Avenue, Lambayeque, 14013 Perú Peru; 3 Universidad Continental, 355 Junin Street, Miraflores, 15046 Lima, Perú

**Keywords:** Costello syndrome, Infant, HRAS gene, Leukodystrophy, Multisystem involvement

## Abstract

**Background:**

Costello syndrome (CS) is a rare genetic condition characterized by dysregulation of the signaling pathway, phenotypic alteration due to fetal macrosomia or growth retardation, facial abnormalities, loose skin, cardiovascular abnormalities, and a variable degree of intellectual disability.

**Case presentation:**

We describe the case of a 20-month-old male patient with fetal macrosomia and polyhydramnios, presenting psychomotor development delay and growth limitation during the first months of life. CS was diagnosed at four months of age after detecting a variant of the HRAS gene c.35G > C (p.G12A). A clinical description of his condition was recorded throughout his life, including cardiovascular diseases, endocrine disorders, and recurrent infections. At 20 months of age, after presenting events of marked hypotonia and generalized seizures, brain magnetic resonance revealed symmetrical lesions of the infra- and supratentorial white matter in both cerebral hemispheres, which resulted in the diagnosis of cerebral leukodystrophy. The patient had a rapid and progressive deterioration that eventually led to death.

**Conclusions:**

This is the first report of a case of CS in Peru. In addition, this is a case that presented with multisystemic conditions culminating in leukodystrophy, which is a rare event according to the literature.

## Background

Costello syndrome (CS, OMIM #218040) is a rare genetic condition discovered in 1971 by Dr. Jack Costello, a New Zealand pediatrician [1], who firstly recorded the cases of two adequate-weight newborns with feeding difficulties and subsequent growth retardation, coarse facial features, and cardiovascular abnormalities [2]. Activation of the HRAS germline mutations along with its ubiquitous expression on the chromosome 11p15 causes CS to be classified as an autosomal dominant RASopathy, a group of genetic conditions in which the RAS signaling pathway is dysregulated [3]. These genomic alterations give rise to different phenotypic traits in other conditions such as the cardio-facio-cutaneous (CFC), Noonan, and Noonan with multiple lentigines syndromes [3], and result in neoplasms [4]. Patients may exhibit a sociable and friendly personality despite having an intellectual deficit of varying degrees [2]. Structural findings in the central nervous system include absolute or relative macrocephaly, ventricular dilatation, and posterior fossa crowding whose severity may meet the criteria for the Chiari malformation type 1 [4].

Leukodystrophies constitute a heterogeneous group of disorders with highly variable clinical manifestations and pathological mechanisms [5]. The prevalence of leukodystrophies among the world population is low (1/100,000 newborns) [3]. Currently, there are more than 107 diseases identified as leukodystrophies that belong to different categories such as proximal or hypomyelinating leukodystrophies, lysosomal storage or cavitary-type conditions, and unclassified or undetermined diseases. Each leukodystrophy is the result of a defect in one of the genes that control myelin production [6] and usually becomes evident during the first months of life, accompanied by hypotonia, which gradually turns into spastic diplegia or tetraplegia, developmental delay, seizures, ataxia, and dyskinesia [7]. In later stages, patients experience problems in swallowing and breathing, triggering a fatal outcome in most cases [8]. Treatment for most leukodystrophies is symptomatic. It includes medication as well as physical, occupational, and speech therapies, which is supported by nutritional, educational, and recreational programs [6].

The interest of this case lies in the timely recognition of CS due to cardiovascular complications [9] and an increased tumor risk found after neoplasm screening for rhabdomyosarcoma [10].

## Case presentation

We present the case of a 20-month-old male patient who was the result of a fourth pregnancy of a 30-year-old mother and a 38-year-old father. The patient’s mother had a controlled pregnancy (more than six prenatal controls). The patient’s parents had no consanguinity relationship nor a history of CS. However, the mother had a history of arterial hypertension, diabetes mellitus, and osteogenesis imperfecta. Since the infant presented fetal macrosomia and polyhydramnios, he was delivered via cesarean section. The Apgar scores at 1 and 5 min after birth were 8 and 9, respectively, with no complications that required hospital admission. The Somatometric index evaluation recorded a weight of 3910 g and a length and head circumference of 51 and 38 cm, respectively.

**Fig. 1:**
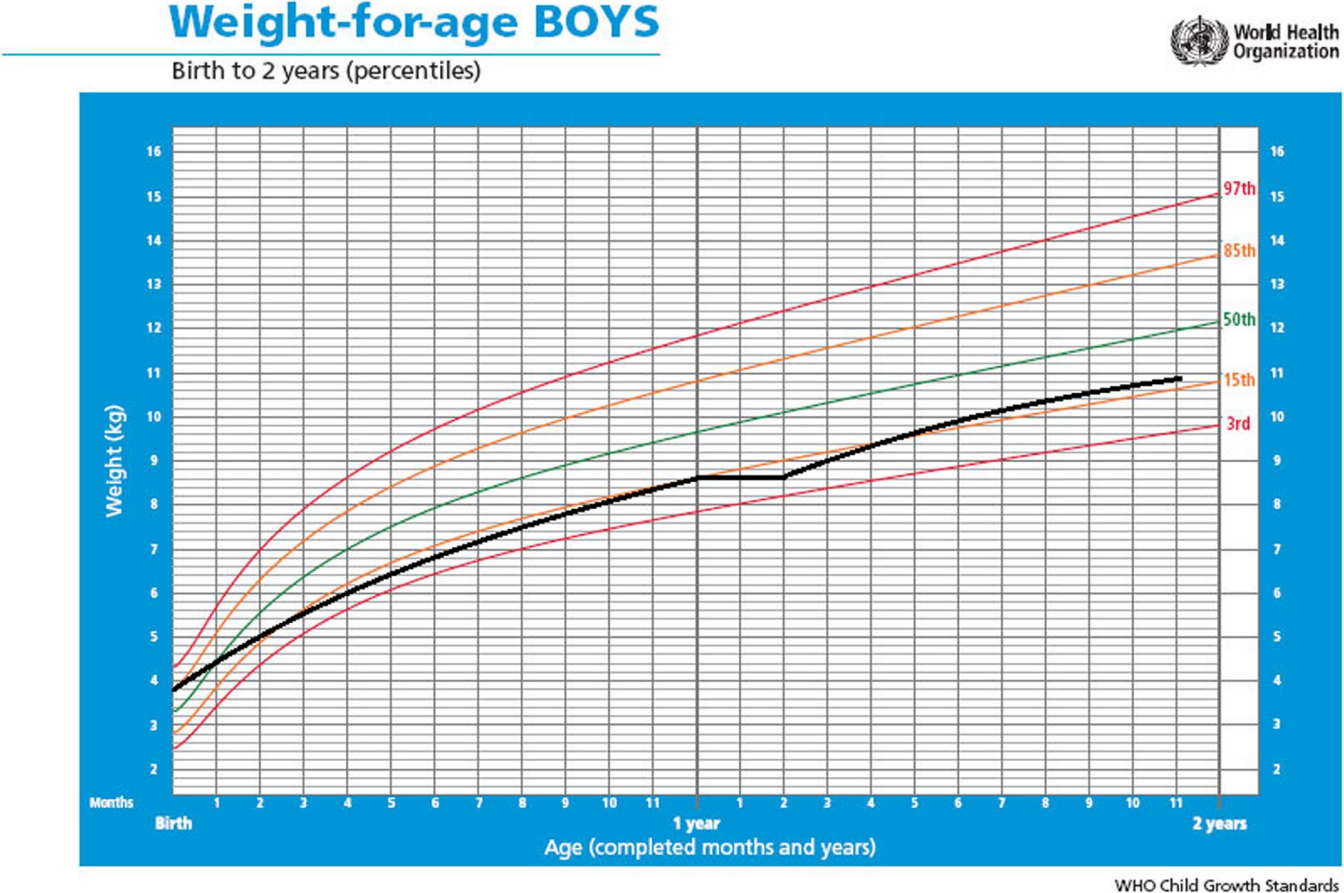
Weight-for-age centile values for the patient with Costello syndrome
- At four months of age, the diagnosis of CS was confirmed by a medical geneticist who reported by exome sequencing tests a variant of the HRAS gene c.35G > C (p.G12A). The patient presented phenotypic features of coarse facial features, skull-face disproportion, full cheeks, wide and depressed nasal bridge, anteverted nostrils, increased palmar and plantar creases, and curved, low-set, posteriorly rotated ears. Audiometry evaluation showed mild bilateral hearing loss and eye fundus examination revealed delayed myelination in both eyes.
- At five months of age, the patient was diagnosed with hypertrophic cardiomyopathy, mitral regurgitation, and diastolic dysfunction. He was administered Verapamil 8 mg every 8 h to treat the hypertrophic cardiomyopathy.
- At one year of age, he was diagnosed with hypothyroidism and treated with levothyroxine 25 mg every 24 h. At 14 months of age, the patient had a weight of 8.6 kg (under the 15th percentile), a length of 74 cm (under the 10th percentile), and his vital signs were within normal ranges. Physical examination revealed macrocephaly, nystagmus (visual disturbance), the aforementioned cranial characteristics along with thick lips, epicanthal folds, large pinnae, thick eyebrows, dental malocclusion, and strabismus (Fig. 2A and C).

The chronological clinical evolution of the patient is detailed below:

- The patient was diagnosed with right cryptorchidism five days after birth. After one month and 16 days of life, he presented with emesis that occurred after breastfeeding. The infant was subsequently diagnosed with hypertrophic pyloric stenosis, which was surgically managed via a Fredet-Ramstedt pyloromyotomy.
- At two months of age, psychomotor development retardation was detected, and the patient’s growth curve was in the 15th percentile due to limited increase in weight and length (Fig. 1, blue line).

**Fig. 2:**
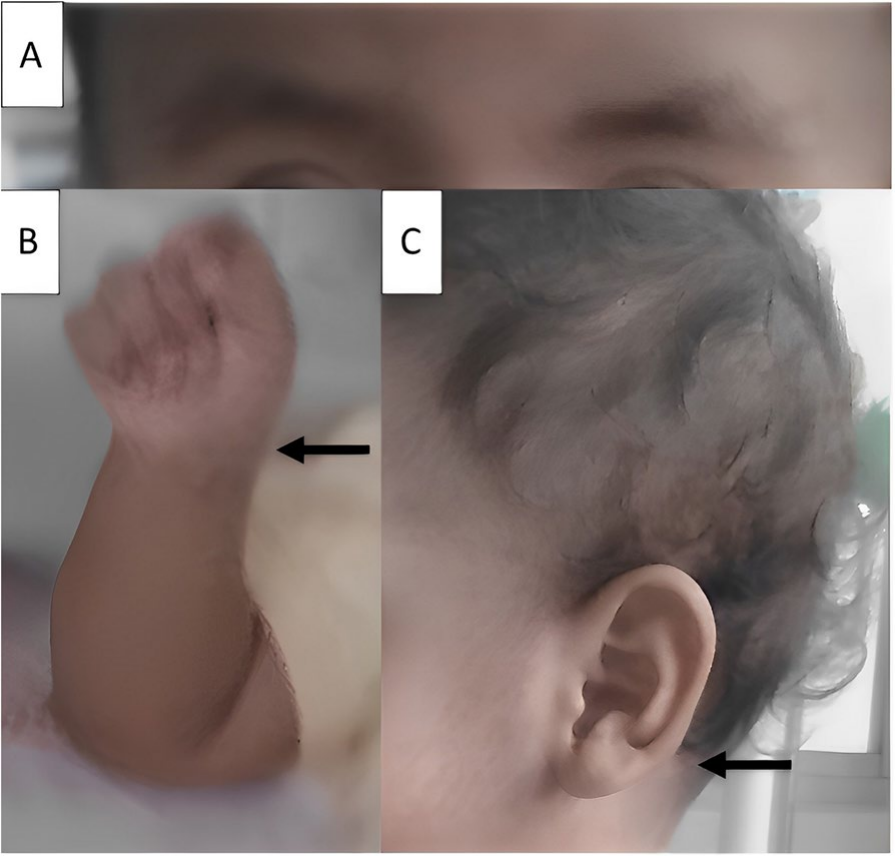
Photograph of the patient showing facial appearance of Costello syndrome. **A** Thick eyebrows. **B** Ulnar deviation of hands. **C** Low-set ear, large pinna
- The musculoskeletal evaluation revealed a short neck, hypotonia, increased anteroposterior chest diameter, broad distal phalanges, ulnar deviation of the hands (Fig. 2B), and pectus carinatum. Skin assessment based on epithelial alterations revealed loose skin of the hands and feet, curly hair, and deep palmar and plantar folds.
- At 20 months of age (14 days before admission), the patient presented with a typical clinical condition of viral illness and received symptomatic treatment for loose stools and a cold. His mother stated that seven days before admission, the patient presented with altered visual acuity (accidentally bumping into walls and objects), and five days before admission, she observed marked hypotonia. The patient was referred to a pediatric neurologist and received treatment, though he showed no improvement.
- Upon hospital admission, the patient presented generalized seizures and aspiration pneumonia, and was consequently admitted to the pediatric intensive care unit. The brain magnetic resonance imaging revealed symmetrical lesions of the infra- and supratentorial white matter in both cerebral hemispheres: the centra semiovale, periventricular white matter and the corpus callosum (front and posterior part). No morphological or brain parenchymal signal-intensity abnormalities were detected nor any alterations in the cerebellar hemispheres, cerebellopontine angles, thalamus, basal ganglia, pituitary gland, eyeballs, or optic nerves (see Fig. 3).
- The patient was diagnosed with cerebral leukodystrophy of unknown etiology. Etiology tests for leukodystrophy were not possible due to the negative prognosis and progressive deterioration and the patient died.
-
  **Fig. 3:**
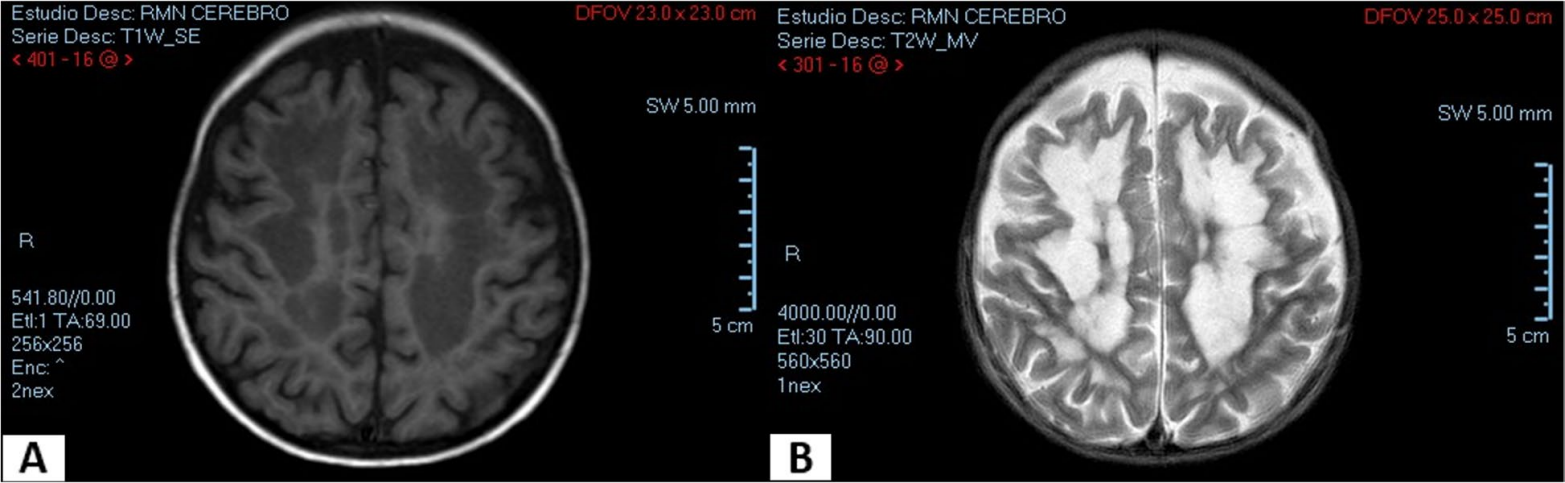
Magnetic resonance imaging. Cross-sectional area of the brain of the patient with Costello syndrome. Infra and supratentorial white matter involvement. **A** T1-weighted sequence. **B** T2-weighted sequence

## Discussion and conclusions

Since the discovery of the genetic cause of CS in 2005 [2] and until diagnosis was determined to be based on clinical findings and confirmed by testing the *HRAS* gene mutation [9, 11], approximately 250 cases had been reported until 2012 [9], which increased to 300 cases by 2014 [12]. By 2019, the prevalence of this syndrome was estimated to be close to 1 case per 300,000 live births [4]. There may be more cases, but being an unfamiliar and infrequently reported syndrome, there are no exact data available, which may result in CS being underdiagnosed.

The patient presented with genotypic and phenotypic data of CS such as fetal macrosomia and growth retardation, characteristic facial abnormalities and loose skin [4, 9, 13], accompanied by cardiovascular abnormalities and other alterations. Additionally second most common mutation is p.G12A which is highly associated with increased chances of malignancies [11].

Based on the child growth standards of the World Health Organization, he was in the 85th percentile at birth, and under the 15th percentile at the age of 14 months. Therefore, he was diagnosed with growth retardation, which is a criterion defined by Costello when reporting the first case of CS in 1971 [1].

Approximately 80% of cases report polyhydramnios during pregnancy and a high birth weight, followed by neonatal hypoglycemia [3, 10, 11]. In our case, the mother had a history of polyhydramnios during pregnancy and her previous children had a high birth weight (85th percentile).

In 2– 4-month newborns presenting with emesis, hypertrophic stenosis of the pylorus is common [4, 9]. This is consistent with our report in which the patient presented with food vomiting a few days after starting breastfeeding, and was diagnosed with hypertrophic pyloric stenosis.

The skin of the palms and soles of the feet were loose, with thick folds, in addition to curly hair and abundant eyebrows, all features typical of CS [4]. Cutaneous papillomas, acanthosis nigricans, and hyperpigmentation may also be present [4]. While patients with CFC syndrome also have curly hair, their eyebrows are typically sparse or absent [14].

Congenital disorders such as neonatal hypothyroidism have been described in a few CS cases [15], and more frequently in patients with Noonan [16] and LEOPARD syndromes. Cryptorchidism has rarely been reported [15], which is not the case for Noonan syndrome, where it is very common. Ulnar deviation of the wrists has been described in CS with a high incidence (63%) [17], unlike other congenital syndromes, where it has not been reported.

Cardiovascular involvement is present in two of every three patients with CS. The patient in our report presented hypertrophic cardiomyopathy, which has been occasionally reported in North American, European, and Asian countries [2, 4, 12, 18]. Heart function monitoring is recommended after diagnosis, through electrocardiogram, echocardiogram, 24-hour Holter monitoring, and strict follow-up due to the multiple complications that may occur.

Many patients with CS develop malignant tumors, such as mainly neuroblastoma and rhabdomyosarcoma [4, 19]. Until our patient reached the age of 14 months, he did not present any malignant tumor.

Cerebral leukodystrophy is the progressive degeneration of the white matter of the brain due to imperfect growth or development of the myelin sheath [5]. This condition has never been reported in a patient with CS before. However, in our reported case, it occurred in a progressive, rapid, and very aggressive manner with a fatal outcome.

CS is characterized by many multisystemic affections and has no specific treatment. Therefore, treatment should be symptomatic and multidisciplinary. Enteral feeding by nasogastric tube is required from the very beginning or during the first days. Patients may benefit from a symptomatic treatment based on papilloma management, strabismus and myopia correction, speech therapy, occupational therapy, and psychomotricity and physical therapy for joint and postural abnormalities, especially to improve their quality of life [4, 19, 20]. For the early detection of possible tumors, assessment with abdominal pelvic ultrasound every 3–6 months is recommended until the end of puberty. Prognosis depends on the severity of the disease and the development of malignant tumors, where our case presented with aggressive cerebral leukodystrophy.

Based on the evaluation by a pediatric neurologist and a geneticist, Costello’s syndrome was initially diagnosed. However, the clinical manifestations in common with leukodystrophy (demyelination, macrocephaly, and rapidly progressive neurological deterioration that affects vision, auditory processing, and motor skills) have probably masked the evolution of CS. Both underlying conditions have been suggested to be responsible for the patient’s status, as they share the structural damage at the oligodendrocyte level, specifically in the connexin proteins 32 and 47 [21], which are responsible of central nervous system myelination.

A complete screening of patients with CS is recommended, not only at the cardio-facial level, as it has been commonly reported, but also at the endocrine and neurological levels.

## Data Availability

All data generated or analysed during this study are included in this published article.

## Abbreviations

CFC: Cardio-facio-cutaneous
CS: Costello syndrome
LV: Left ventricle
